# Identification of 67 Pleiotropic Genes Associated With Seven Autoimmune/Autoinflammatory Diseases Using Multivariate Statistical Analysis

**DOI:** 10.3389/fimmu.2020.00030

**Published:** 2020-02-03

**Authors:** Xiaocan Jia, Nian Shi, Yu Feng, Yifan Li, Jiebing Tan, Fei Xu, Wei Wang, Changqing Sun, Hongwen Deng, Yongli Yang, Xuezhong Shi

**Affiliations:** ^1^Department of Epidemiology and Biostatistics, College of Public Health, Zhengzhou University, Zhengzhou, China; ^2^Department of Physical Diagnosis, The First Affiliated Hospital of Zhengzhou University, Zhengzhou, China; ^3^Department of Occupational and Environmental Health, College of Public Health, Zhengzhou University, Zhengzhou, China; ^4^Center for Bioinformatics and Genomics, School of Public Health and Tropical Medicine, Tulane University, New Orleans, LA, United States

**Keywords:** metaCCA, autoimmune/autoinflammatory diseases, pleiotropic gene, GWAS, shared gene

## Abstract

Although genome-wide association studies (GWAS) have a dramatic impact on susceptibility locus discovery, this univariate approach has limitations in detecting complex genotype-phenotype correlations. Multivariate analysis is essential to identify shared genetic risk factors acting through common biological mechanisms of autoimmune/autoinflammatory diseases. In this study, GWAS summary statistics, including 41,274 single nucleotide polymorphisms (SNPs) located in 11,516 gene regions, were analyzed to identify shared variants of seven autoimmune/autoinflammatory diseases using the metaCCA method. Gene-based association analysis was used to refine the pleiotropic genes. In addition, GO term enrichment analysis and protein-protein interaction network analysis were applied to explore the potential biological functions of the identified genes. A total of 4,962 SNPs (*P* < 1.21 × 10^−6^) and 1,044 pleotropic genes (*P* < 4.34 × 10^−6^) were identified by metaCCA analysis. By screening the results of gene-based *P*-values, we identified the existence of 27 confirmed pleiotropic genes and highlighted 40 novel pleiotropic genes that achieved statistical significance in the metaCCA analysis and were also associated with at least one autoimmune/autoinflammatory in the VEGAS2 analysis. Using the metaCCA method, we identified novel variants associated with complex diseases incorporating different GWAS datasets. Our analysis may provide insights for the development of common therapeutic approaches for autoimmune/autoinflammatory diseases based on the pleiotropic genes and common mechanisms identified.

## Introduction

Autoimmune/autoinflammatory diseases are chronic conditions initiated by loss of immunological tolerance to self-antigens (1). In Europe and North America, these conditions occur with an estimated incidence of ~90 cases per 100,000 person-years and a prevalence of between 7.6 and 9.4% (2, 3). The chronic nature of such diseases has a significant impact in terms of the utilization of medical care, direct and indirect economic costs and quality of life. In addition, extensive clinical and epidemiologic observations have shown that autoimmune/autoinflammatory diseases are characterized by familial clustering of multiple diseases, epidemiological co-occurrence, overlapped autoantibody level, and the efficacy of therapies across diseases. These observations provide evidence that different autoimmune/autoinflammatory diseases share a substantial portion of their pathobiology and genetic predisposition underlies disease susceptibility (4–6).

The genetic effect of a single nucleotide polymorphism (SNP) or gene on two or more phenotypic traits can be described as pleiotropic and the outcome is genetically correlated. In general, this concept concerns across-trait architecture (7). To date, 186 statistically significant susceptibility loci have been identified in genome-wide association studies (GWAS) of autoimmune/autoinflammatory diseases (2, 8). These were confirmed in subsequent studies, with more than half of the susceptibility genes found to be shared by at least two distinct autoimmune/autoinflammatory diseases (9–12). For example, *PTPN22* c.1858C>T (rs2476601) was identified as a susceptibility gene in independent GWAS across multiple autoimmune/autoinflammatory diseases (13). In addition, there is evidence that loci associated with predisposition to one disease can have effects on the risk of a second disease, although the risk alleles for the two diseases may not be the same (9). However, the evidence for specific shared risk variants is modest, and consequently the genetic mechanisms underlying the patterns of disease aggregation remain unclear. It is, therefore, important to identify shared genetic risk factors acting through common biological mechanisms and to assess the overlapping pathophysiological relationships among autoimmune/autoinflammatory diseases using effective analytical approaches.

GWAS is a standard univariate approach used to investigate and identify potentially causal or risk-conferring genetic variants associated with common human diseases at the level of individual measurements (14, 15). GWAS, especially those with large sample size, and meta-analysis of multiple studies, have a dramatic impact on susceptibility locus discovery and in addition, highlight and extend the previously observed commonality among autoimmune/autoinflammatory diseases. However, with the identification of millions of SNPs and a growing number of phenotypes, this univariate approach has been used to detect complex genotype-phenotype correlations with limited success. Furthermore, studies of statistical methods have confirmed that multivariate analysis provides higher statistical power for the detection of unexplained heritability due to the consideration of correlations not only among multiple SNPs, but also among different traits or diseases (16). In previous studies, bivariate analysis has been used to investigate genetic risk factors associated with complex traits, and multivariate analysis of this aspect of complex diseases is rare (17, 18). Therefore, multivariate analysis of the publicly available GWAS summary statistics in particular is highly essential and relevant to the identification of pleiotropic genes.

Cichonska et al. (19) recently employed meta-analysis using canonical correlation analysis (metaCCA) to allow multivariate representation of both genotypic and phenotypic variables based on the published univariate GWAS summary statistics. This new approach has been applied to identify potential pleiotropic genes associated with lipid-related measures, psychiatric disorders, and chronic diseases (19–22). In this study, the genetic pleiotropy-informed metaCCA method was used to identify shared variants and pleiotropic effects in seven autoimmune/autoinflammatory diseases: celiac disease (CEL), inflammatory bowel disease (IBD), which includes Crohn's disease (CRO) and ulcerative colitis (UC), multiple sclerosis (MS), primary biliary cirrhosis (PBC), rheumatoid arthritis (RA), systemic lupus erythematosus (SLE) and type 1 diabetes (T1D). In addition, gene-based association analysis was used to refine pleiotropic genes. GO term enrichment analysis and protein-protein interaction network analysis were applied to explore the potential biological function of the identified genes.

## Materials and Methods

### GWAS Datasets

All the GWAS summary statistics of seven autoimmune/autoinflammatory diseases investigated in the present study were downloaded from ImmunoBase (website: https://www.immunobase.org/), which is a web-based resource focused on the genetics and genomics of immunologically-related human diseases. The CEL data were obtained from a second-generation GWAS of 4,533 cases and 10,750 control subjects including 523,402 SNPs (23). The association summary statistics of IBD, including 9,735,446 imputed SNPs, were obtained from a meta-analysis with a total sample size of 59,957 subjects (24). The MS dataset consisted of 464,357 genotyped or imputed SNPs from a collaborative GWAS involving 9,772 cases and 17,376 controls of European descent collected by 23 research teams from 15 different countries (25). The PBC dataset including 1,134,141 SNPs were obtained from a meta-analysis (2,764 cases and 10,475 controls) and an independent cohort study (3,716 cases and 4,261 controls) (26). The RA dataset was also obtained from a GWAS meta-analysis of 5,539 autoantibody-positive RA cases and 20,169 controls of European descent, followed by replication in an independent set of 6,768 RA cases and 8,806 controls, which included a total of 8,254,863 SNPs (27). The SLE dataset comprised 7,219 cases and 15,991 controls of European ancestry, yielding a total of 7,915,250 SNPs from a new GWAS, meta-analysis of published GWAS and a replication study (28). The T1D dataset consisting of 8,781,607 SNPs was extracted from a Mendelian randomization analysis with 5,913 T1D cases and 8,828 reference samples (29). All the samples in the present study came from Northern and Western European ancestry (CEU) population. The summary statistics have been undertaken genomic control in the individual study or meta-analysis. Furthermore, the ImmunoBase was searched using a global cutoff for minor allele frequency (MAF) <99% for all datasets. Further details of the inclusion criteria and quality control methods used in the different GWAS studies are described in the original publications (23–29). It should be noted that the summary statistics of GWAS or meta-analysis were required to include *P*-values, regression coefficients and standard error.

### Data Preparation

When dealing with the various datasets, we first combined the seven summary statistics for the common SNPs studied in all datasets. The result of 324,031 overlapping SNPs was then selected to for multivariate analysis. Second, gene annotation was completed according to the 1,000 Genome datasets (website:https://www.cog-genomics.org/static/bin/plink/glist-hg19) using PLINK1.9. Third, the linkage disequilibrium (LD)-based SNP pruning method was used to remove SNPs with large pairwise correlations. This SNP pruning method was performed using 50 SNPs as a window where the LD was calculated between each pair of SNPs. The MAF was also used as a criterion in the SNP pruning method to remove the SNPs with smaller MAFs for pairs with *R*^2^ > 0.2. Following this initial removal of SNPs in high LD, each sliding window of five SNPs forward and the process was repeated until there were no pairs of SNPs with MAFs > 0.2 (30). All datasets were pruned using the HapMap 3 CEU genotypes as a reference panel (website: http://www.sanger.ac.uk/resources/downloads/human/hapmap3.html). Following this pruning procedure, 41,274 SNPs located in 11,516 gene regions remained and were included in the metaCCA analysis. Finally, the regression coefficient beta was normalized before conducting the metaCCA analysis in instance when the individual-level dataset genotype and phenotype matrices were not standardized. Standardization was achieved subsequently according to the following equation:

(1) $$\begin{array}{r} \beta_{gp}^{STANDR}=\frac{1}{\sqrt{n}SE_{\text{gp}}}\times \beta_{\text{gp}} \end{array}$$

where *SE*_*gp*_ is the standard error of β_*gp*_, as given by the original GWAS result, *g* is the number of genotypic variables, *p* is the number of phenotypic variables, and *n* is the sample number for each disease.

### MetaCCA Analysis

MetaCCA is an extension of the CCA method, which requires a full covariance matrix (∑), consisting of four covariance matrices, which can be obtained using the following formula:

(2) $$\sum =\left(\begin{array}{cc} \sum^{\wedge }XX & \sum XY\\ \;\sum_{XY}^{T} & \sum^{\wedge }YY \end{array}\right)\;$$

where ∑*XY* is a cross-covariance matrix between all genotypic and phenotypic variables, $\sum^{\wedge }XX$ is a genotypic correlation structure between SNPs, $\sum^{\wedge }YY$ is a phenotypic correlation structure between traits, and $\sum_{XY}^{T}$ is the transposition of ∑*XY* (19). ∑*XY* is constructed as the normalized regression coefficient β*gp*:

(3) $$\begin{array}{r} \sum XY=\left(\begin{array}{cccc} \beta_{11} & \beta_{12} & \cdots & \beta_{1p}\\ \beta_{21} & \beta_{22} & \cdots & \beta_{2p}\\ \vdots & \vdots & \ddots & \vdots \\ \beta_{g1} & \beta_{g2} & \cdots & \beta_{gp} \end{array}\right) \end{array}$$

In metaCCA, $\sum^{\wedge }XX$ is calculated using a reference database representing the study population, such as the 1,000 Genomes database, or other genotypic data available for the target population. More accurate results will be obtained if $\sum^{\wedge }XX$ is estimated from the target population or a population of the same ethnicity instead of interracial populations (19). In our study, $\sum^{\wedge }XX$ was estimated using the reference SNP dataset of the HapMap 3 CEU.

The phenotypic correlation structure $\sum^{\wedge }YY$ was computed based on ∑*XY*, with each $\sum^{\wedge }YY$ corresponding to a Pearson correlation coefficient between the vector of β estimates from *p* phenotypic variables across *g* genetic variants. It has been demonstrated that the accuracy of estimate increased with the value of *g*. Thus, $\sum^{\wedge }YY$ values were calculated from summary statistics of 324,031 overlapping SNPs, even if 41,274 of them were used for subsequent analysis.

We next determined whether the full covariance matrix was positive semidefinite (PSD); if not, an iterative procedure was used to shrink the full covariance matrix until ∑ became PSD. In the next analysis, the PSD of the full covariance matrix was entered into the CCA framework to determine the final genotype-phenotype association (19), where the correlation between genotype and phenotype is defined as the canonical correlation, *r* (31).

In this study, two types of multivariate analysis were evaluated and the conservative Bonferroni correction method was used as the threshold for nominal significance. If the *P*-value of the canonical correlation r of any SNP was smaller than 1.21 × 10^−6^ (=0.05/41,274), it was deemed significantly associated with the seven diseases. Second, multivariate SNP-multivariate phenotype association analysis at the gene level was performed to identify any potential pleiotropic gene. Similarly, genes with a *P*-value of canonical correlation smaller than 4.34 × 10^−6^ (=0.05/11,516) were identified as potential pleiotropic genes associated with multiple autoimmune/autoinflammatory diseases.

### Gene-Based Association Analysis

VEGAS2 (Versatile Gene-based Association Study−2) is a method of gene-based association analysis used to calculate the correlation of multiple SNPs in a gene region with one phenotype using original GWAS summary statistics (32). This method has been widely applied in the genetics studies and has been shown to offer higher sensitivity and lower false positive rates compared to other gene-based approaches (33). In the present study, the VEGAS2 method was combined with the metaCCA to refine the genes identified using the metaCCA by computing the gene-based *P*-value for each specific disease. This analysis was performed using https://vegas2.qimrberghofer.edu.au/, with a threshold of 1.00E-06.

### GO Term Enrichment Analysis

In clarifying polygenic associations, it is useful to determine whether or not the implicated genetic variants occur in genes involved in a biological pathway (34). GO term enrichment analysis classifies gene functions based on three main categories: molecular function, cellular component and biological process. We conducted GO term enrichment analysis to determine which GO term were over- or under-represented). All significant genes re-identified by VEGAS2 in our study were annotated and enriched (website: http://amp.pharm.mssm.edu/Enrichr/). An adjusted *P* < 0.05 in the enrichment analysis was considered to indicate nominal significance (35).

### Protein-Protein Interaction Network

Protein-protein interactions (PPIs) are crucial for all biological processes and the networks provide many new insights into protein function (36, 37). In order to detect interactions and associations of all putative pleiotropic genes, PPIs analysis were conducted by searching the Search Tool for the Retrieval of Interacting Genes/Proteins (STRING) database (website: http://string-db.org/), which comprises known and predicted associations from curated databases or high-throughput studies, in addition to other associations derived from text mining, co-expression, and protein homology (38).

## Results

### Potential Pleiotropic SNPs and Genes Identified by metaCCA Analysis

**Figure 1:**
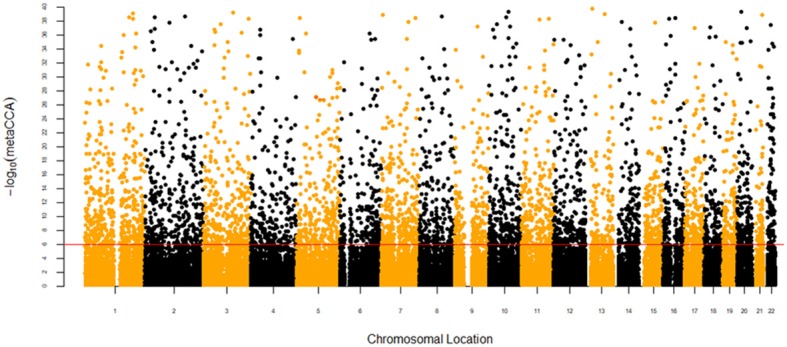
Manhattan plot of –log_10_(metaCCA) values for univariate SNP-seven autoimmune/autoinflammatory diseases analysis. The red line marks the –log_10_(metaCCA) value of 5.92 corresponding to *P* < 1.21 × 10^−6^. If the –log_10_(metaCCA) value of a certain SNP was >5.92, this SNP was identified as a pleiotropic SNP for seven diseases.

After gene annotation and SNP pruning, 41,274 SNPs located in 11,516 gene regions were available for the metaCCA analysis. The size of SNP representation among the genes ranged from 1 to 213 SNPs, and the median number of SNPs in each gene was 3.72. For the univariate SNP-multivariate phenotype analysis, 4,962 SNPs reached the Bonferroni corrected threshold (*P* < 1.21 × 10^−6^), and the canonical correlation *r* between each SNP and phenotype ranged from 0.0372 to 0.6586. The results are presented in a Manhattan plot in Figure 1. For the multivariate SNP-multivariate phenotype analysis, 1,044 genes with a significance threshold of *P* < 4.34 × 10^−6^ were identified as potential pleiotropic genes. The canonical correlation r between genotype and phenotype ranged from 0.0322 to 0.5899.

### Refining the Pleiotropic Genes by Gene-Based Association Analysis

The VEGAS2 algorithm based on original GWAS summary statistics was used to identify association of one gene with a specific disease. In the gene-based association analysis, 19 genes were identified for CEL, 111 for IBD, 16 for MS, 20 for PBC, 19 for RA, 20 for SLE, and 33 significant genes for T1D (*P* = 1.00E-06).

By screening the results of gene-based analysis *P*-values, we identified 67 putative pleiotropic genes yielding statistical significance in the metaCCA analysis and found to be associated with at least one disease in the VEGAS2 analysis. In particular, 17 genes were found to be associated with more than one disease in the original GWAS. The results of the metaCCA and VEGAS2 analysis are summarized in Table 1.

**Table 1:** The 67 pleiotropic genes identified by the metaCCA and VEGAS2 analysis.

**Locus**	**Gene**	**MetaCCA *P*-value**	**VEGAS** ***P*****-value**						
			**CEL**	**IBD**	**MS**	**PBC**	**RA**	**SLE**	**T1D**
1	*ADAD1*^a^	1.05E-30	1.00E-06	1.00E-06	0.29	1.23E-02	1.10E-05	2.58E-02	6.97E-03
2	*ADCY5*	1.44E-06	2.40E-02	3.91E-02	1.00E-06	3.75E-02	1.29E-02	1.32E-02	0.87
3	*AHI1*	2.03E-87	5.05E-02	0.30	1.00E-06	0.36	0.54	1.94E-03	4.75E-02
4	*ATG5*	1.53E-16	0.33	7.00E-06	0.53	1.87E-03	1.54E-03	1.00E-06	0.94
5	*C1orf106*	1.82E-33	0.09	3.09E-02	7.79E-02	1.22E-02	1.00E-06	5.30E-05	3.73E-02
6	*C1orf141*^a^	7.70E-11	0.44	1.00E-06	0.87	1.00E-06	9.85E-03	2.93E-03	0.53
7	*C5orf56*	1.87E-16	2.07E-03	1.00E-06	0.29	0.67	9.41E-02	1.86E-02	6.48E-02
8	*CALU*^a^	2.10E-10	0.20	1.75E-02	2.21E-02	1.00E-06	1.00E-06	1.00E-06	7.18E-02
9	*CCDC136*^a^	3.51E-25	0.030	2.90E-02	0.38	1.00E-06	1.00E-05	1.00E-06	0.39
10	*CD58*	5.43E-13	0.520	2.44E-02	0.158	0.71	1.00E-06	0.60	6.64E-02
11	*CIITA*^a^	4.85E-28	4.05E-03	0.21	1.00E-06	1.00E-06	0.54	1.47E-04	1.00E-06
12	*CLEC16A*^a^	9.26E-85	1.28E-02	2.12E-03	1.00E-06	1.00E-06	0.62	1.00E-06	1.00E-06
13	*CUL2*	2.56E-19	0.40	1.00E-06	0.39	2.91E-02	0.78	4.12E-04	5.76E-02
14	*CUX2*	2.28E-34	0.45	7.00E-06	0.119	2.60E-03	3.06E-02	0.12	1.00E-06
15	*DEAF1*	7.99E-14	0.40	9.22E-02	0.85	9.31E-02	0.86	1.00E-06	0.42
16	*DGKQ*	1.61E-15	0.88	0.43	2.18E-03	1.00E-06	9.95E-02	4.57E-03	0.32
17	*DNMT1*	6.97E-07	0.39	6.87E-04	7.82E-04	2.00E-06	5.03E-04	1.00E-06	9.33E-02
18	*EFR3B*^a^	1.70E-99	0.34	1.00E-06	2.72E-02	1.00E-06	2.78E-03	0.47	1.54E-02
19	*ERAP2*	1.77E-49	0.98	1.00E-06	0.25	6.88E-04	0.45	0.57	4.76E-02
20	*EVI5*	4.08E-19	0.39	0.23	1.00E-06	0.65	1.18E-02	0.23	1.50E-02
21	*FGF2*^a^	1.78E-08	1.00E-06	1.00E-06	9.08E-03	0.39	3.04E-03	0.18	1.40E-05
22	*FYCO1*	2.73E-11	1.00E-06	2.45E-02	1.00E-02	0.74	0.49	0.57	0.86
23	*GRIP1*	2.20E-62	1.39E-02	1.00E-06	0.20	0.25	0.25	4.43E-02	0.73
24	*HNF1B*	2.17E-10	0.14	5.60E-03	0.44	0.11	2.94E-02	0.31	1.00E-06
25	*IKZF1*	4.37E-10	0.02	0.42	7.70E-02	0.29	4.02E-04	0.49	1.00E-06
26	*IKZF3*	2.57E-203	0.41	1.00E-06	9.31E-02	0.47	0.43	0.54	2.63E-02
27	*IL22RA2*	1.55E-11	0.19	0.46	1.00E-06	2.85E-03	0.67	3.28E-02	0.12
28	*IL23R*^a^	1.00E-232	0.90	1.00E-06	0.45	1.00E-06	0.11	8.16E-02	0.91
29	*INPP1*	3.40E-06	0.35	0.25	0.695	4.30E-05	3.65E-03	1.00E-06	0.56
30	*IRF1*	6.56E-72	9.87E-03	1.00E-06	4.58E-02	0.42	3.38E-02	0.53	5.98E-02
31	*ITGAM*	2.41E-48	5.22E-02	0.80	7.03E-02	0.17	0.20	1.00E-06	0.23
32	*JAK2*	6.34E-118	0.18	1.00E-06	2.21E-03	0.63	0.40	8.33E-02	0.53
33	*KIAA1109*	5.27E-16	2.00E-06	1.00E-05	1.79E-03	0.12	4.27E-02	0.35	1.00E-06
34	*LINC00271*	1.22E-08	1.63E-02	0.97	1.00E-06	0.48	0.26	1.16E-03	1.78E-03
35	*LOC101927051*^a^	5.60E-11	0.55	1.00E-06	0.13	1.00E-06	3.00E-06	4.24E-04	4.84E-03
36	*LOC285626*	3.83E-97	0.50	1.00E-06	5.05E-02	0.40	0.74	0.94	0.51
37	*LTF*	5.48E-09	1.00E-06	0.17	0.33	0.76	5.55E-02	0.93	1.51E-04
38	*MACROD2*	9.46E-07	0.35	4.60E-05	2.91E-02	1.00E-06	6.85E-03	0.12	1.88E-02
39	*MAGI3*^a^	7.55E-24	0.65	1.10E-02	0.27	0.81	1.00E-06	8.90E-05	1.00E-06
40	*MAP3K7*	2.77E-66	0.30	1.00E-06	0.31	7.85E-02	0.25	0.48	0.46
41	*MAP4K4*^a^	3.30E-24	1.00E-06	1.00E-06	0.15	5.08E-02	0.73	9.30E-03	7.47E-02
42	*MPZL3*^a^	5.37E-09	1.45E-02	0.86	6.22E-04	1.00E-06	1.00E-06	1.45E-03	0.18
43	*MST1R*	3.72E-37	0.93	1.00E-06	0.56	0.4588	0.84	1.27E-02	5.08E-02
44	*NSD1*	1.16E-25	0.55	1.00E-06	1.00E-05	0.16	1.42E-02	0.55	1.44E-02
45	*PAPOLG*^a^	7.00E-08	4.00E-06	1.00E-06	1.07E-02	0.11	1.00E-06	4.40E-02	0.50
46	*PLCL2*	2.97E-17	0.18	0.22	1.50E-02	3.00E-06	1.00E-06	0.62	0.72
47	*PRKAA1*	4.54E-18	0.12	1.00E-06	3.90E-02	0.371	0.34	0.12	0.30
48	*PTPN2*^a^	1.06E-09	6.00E-06	1.00E-06	7.66E-03	9.56E-02	1.00E-06	0.15	1.00E-06
49	*PUS10*	5.93E-101	7.00E-06	1.00E-06	1.60E-02	0.40	0.20	0.16	7.89E-02
50	*RBM17*^a^	2.49E-25	0.252	3.36E-02	3.10E-05	0.64	1.00E-06	0.47	1.00E-06
51	*RNASET2*	1.22E-08	0.162	1.00E-06	0.99	4.08E-04	1.77E-02	8.93E-02	0.24
52	*SH3BP1*^a^	5.60E-11	0.562	1.00E-06	0.11	1.00E-06	3.00E-06	4.39E-04	4.35E-03
53	*SHISA5*	5.12E-118	3.68E-02	1.00E-06	0.32	0.70	0.60	0.46	0.14
54	*SLC26A4*	1.29E-35	0.90	1.00E-06	2.79E-02	0.17	0.90	0.23	9.07E-02
55	*THADA*	9.70E-149	0.48	1.00E-06	0.30	0.51	0.83	4.70E-02	2.17E-03
56	*TNFAIP3*	6.86E-33	0.48	0.38	1.99E-04	2.60E-03	9.00E-06	1.00E-06	5.88E-02
57	*TNIK*	3.66E-54	1.00E-06	0.198	5.73E-04	8.17E-02	0.34	2.71E-03	8.36E-02
58	*TNIP1*	3.52E-34	0.12	0.158	0.49	4.82E-04	9.01E-04	1.00E-06	0.39
59	*TNS1*	1.56E-18	0.11	1.00E-06	0.64	3.03E-02	8.87E-02	0.88	2.76E-02
60	*TP63*	1.05E-26	1.00E-06	2.00E-06	2.85E-02	0.33	0.68	0.25	7.99E-02
61	*TRPV4*	2.37E-09	3.43E-04	3.74E-02	0.15	1.46E-03	1.13E-03	0.25	1.00E-06
62	*TTC34*	1.98E-09	5.60E-05	1.19E-02	1.00E-06	4.60E-05	1.30E-04	4.65E-02	0.46
63	*TYK2*	1.06E-06	0.11	1.00E-06	3.00E-06	2.80E-05	5.00E-06	7.00E-06	3.82E-03
64	*USP34*	9.72E-217	4.00E-06	1.00E-06	4.59E-02	0.38	1.15E-02	0.40	0.35
65	*WDR78*	4.90E-52	2.04E-03	1.00E-06	0.12	8.53E-03	1.30E-02	0.85	0.76
66	*WNT11*	4.60E-19	0.19	1.00E-06	0.83	0.19	0.33	0.47	0.45
67	*ZNF365*	1.41E-164	0.25	1.00E-06	0.16	0.55	0.12	4.37E-02	0.36

a *This gene was associated with more than one disease in the VEGAS2 analysis*.

Specifically, 27 of these 67 putative pleiotropic genes had previously been reported to be associated with more than one of these seven diseases. Of these 27 confirmed pleiotropic genes, six genes (*ADAD1, CIITA, CLEC16A, IL23R, MAGI3*, and *PTPN2*) were associated with more than one disease in the VEGAS2 analysis of the original GWAS summary statistics. Of the 40 novel putative pleiotropic genes detected, 16 were previously reported to be associated with only one autoimmune/autoinflammatory disease. *EFR3B* and *RBM17* were reported to be associated with T1D only in published studies but were shown to be associated with multiple diseases in the VEGAS2 analysis. The remaining 24 significant genes were implicated as candidate novel pleiotropic genes for these seven diseases. More significantly, nine genes (*C1orf141, CALU, CCDC136, FGF2, LOC101927051, MAP4K4, MPZL3, PAPOLG*, and *SH3BP1*) were associated with more than one disease in the VEGAS2 analysis, although they had never been reported to be associated with any autoimmune/autoinflammatory disease. The detailed features of 67 significant pleiotropic genes are shown in Table 2.

**Table 2:** The features of 67 significant pleiotropic genes.

**Locus**	**Gene**	**Chr**	**Number of SNPs**	***r*-value**	**MetaCCA *P*-value**	**Gene type**
1	*ADAD1*^a^	4	3	0.08	1.05E-30	Confirmed
2	*ADCY5*	3	11	0.05	1.44E-06	Novel*
3	*AHI1*	6	13	0.12	2.03E-87	Confirmed
4	*ATG5*	6	19	0.05	1.53E-16	Confirmed
5	*C1orf106*	1	14	0.08	1.82E-33	Confirmed
6	*C1orf141*^a^	1	1	0.05	7.70E-11	Novel
7	*C5orf56*	5	12	0.06	1.87E-16	Novel*
8	*CALU*^a^	7	6	0.05	2.10E-10	Novel
9	*CCDC136*^a^	7	2	0.08	3.51E-25	Novel
10	*CD58*	1	8	0.05	5.43E-13	Confirmed
11	*CIITA*^a^	16	8	0.07	4.85E-28	Confirmed
12	*CLEC16A*^a^	16	47	0.10	9.26E-85	Confirmed
13	*CUL2*	10	9	0.06	2.56E-19	Novel*
14	*CUX2*	12	42	0.08	2.28E-34	Novel*
15	*DEAF1*	11	9	0.05	7.99E-14	Novel*
16	*DGKQ*	4	1	0.05	1.61E-15	Novel*
17	*DNMT1*	19	10	0.04	6.97E-07	Novel
18	*EFR3B*^a^	2	11	0.13	1.70E-99	Novel*
19	*ERAP2*	5	11	0.09	1.77E-49	Confirmed
20	*EVI5*	1	15	0.06	4.08E-19	Novel*
21	*FGF2*^a^	4	13	0.06	1.78E-08	Novel
22	*FYCO1*	3	6	0.05	2.73E-11	Novel
23	*GRIP1*	12	67	0.12	2.20E-62	Novel
24	*HNF1B*	17	4	0.06	2.17E-10	Novel*
25	*IKZF1*	7	17	0.05	4.37E-10	Confirmed
26	*IKZF3*	17	2	0.18	2.57E-203	Confirmed
27	*IL22RA2*	6	3	0.04	1.55E-11	Novel*
28	*IL23R*^a^	1	9	0.23	1.00E-232	Confirmed
29	*INPP1*	2	1	0.04	3.40E-06	Novel
30	*IRF1*	5	1	0.11	6.56E-72	Confirmed
31	*ITGAM*	16	8	0.09	2.41E-48	Novel*
32	*JAK2*	9	11	0.14	6.34E-118	Confirmed
33	*KIAA1109*	4	24	0.05	5.27E-16	Confirmed
34	*LINC00271*	6	9	0.04	1.22E-08	Novel*
35	*LOC101927051*^a^	22	8	0.05	5.60E-11	Novel
36	*LOC285626*	5	2	0.12	3.83E-97	Novel
37	*LTF*	3	5	0.04	5.48E-09	Novel
38	*MACROD2*	20	15	0.04	9.46E-07	Novel*
39	*MAGI3*^a^	1	18	0.06	7.55E-24	Confirmed
40	*MAP3K7*	6	2	0.11	2.77E-66	Confirmed
41	*MAP4K4*^a^	2	32	0.07	3.30E-24	Novel
42	*MPZL3*^a^	11	2	0.04	5.37E-09	Novel
43	*MST1R*	3	1	0.08	3.72E-37	Confirmed
44	*NSD1*	5	9	0.07	1.16E-25	Novel
45	*PAPOLG*^a^	2	5	0.04	7.00E-08	Novel
46	*PLCL2*	3	23	0.05	2.97E-17	Confirmed
47	*PRKAA1*	5	3	0.06	4.54E-18	Novel
48	*PTPN2*^a^	18	11	0.05	1.06E-09	Confirmed
49	*PUS10*	2	10	0.13	5.93E-101	Confirmed
50	*RBM17*^a^	10	5	0.06	2.49E-25	Novel*
51	*RNASET2*	6	2	0.04	1.22E-08	Confirmed
52	*SH3BP1*^a^	22	8	0.05	5.60E-11	Novel
53	*SHISA5*	3	2	0.14	5.12E-118	Novel
54	*SLC26A4*	7	2	0.08	1.29E-35	Novel
55	*THADA*	2	50	0.15	9.70E-149	Confirmed
56	*TNFAIP3*	6	2	0.08	6.86E-33	Confirmed
57	*TNIK*	3	44	0.11	3.66E-54	Novel
58	*TNIP1*	5	23	0.07	3.52E-34	Confirmed
59	*TNS1*	2	11	0.07	1.56E-18	Novel
60	*TP63*	3	31	0.07	1.05E-26	Novel
61	*TRPV4*	12	11	0.04	2.37E-09	Confirmed
62	*TTC34*	1	2	0.04	1.98E-09	Confirmed
63	*TYK2*	19	5	0.04	1.06E-06	Confirmed
64	*USP34*	2	11	0.18	9.72E-217	Novel
65	*WDR78*	1	13	0.10	4.90E-52	Novel
66	*WNT11*	11	3	0.06	4.60E-19	Novel*
67	*ZNF365*	10	39	0.17	1.41E-164	Novel*

*Confirmed: This gene was previously reported to be associated with more than one autoimmune/autoinflammatory disease*.

Novel^*^: This gene had been reported to be associated with only one autoimmune/autoinflammatory disease.

*Novel: This gene had never been reported to be associated with any autoimmune/autoinflammatory disease*.

a *This gene was associated with more than one autoimmune/autoinflammatory disease in the VEGAS2 analysis*.

### GO Term Enrichment Analysis

GO enrichment analysis revealed that the biological functions of these pleiotropic genes were involved mainly in the metabolism of lipids. When the 67 pleiotropic genes associated with autoimmune/autoinflammatory diseases were used as the gene sets for the GO term enrichment analysis, several functional terms were identified as being enriched. For the GO biological process and molecular function, there were 63 and 5 terms were identified to be significantly enriched in the development of autoimmune/autoinflammatory diseases, respectively. Details of the top five significant GO terms are shown in Table 3.

**Table 3:** Top five significant GO term enrichment of the 67 pleiotropic genes.

**Term (GO_biological_process)**	***P*-value**	**Adjusted *P*-value**	**Genes**
Positive regulation of gene expression (GO:0010628)	1.41E-06	1.32E-03	*CIITA; DNMT1; PRKAA1; HNF1B; FGF2; GRIP1; CUX2; WNT11; DEAF1; DGKQ; IRF1; NSD1; TP63*
Interleukin-23-mediated signaling pathway (GO:0038155)	4.24E-06	1.98E-03	*IL23R; TYK2; JAK2*
Activation of protein kinase activity (GO:0032147)	1.31E-05	4.09E-03	*PRKAA1; TNIK; JAK2;* *FGF2; MAP3K7; ADCY5; MAP4K4*
Cellular response to cytokine stimulus (GO:0071345)	2.07E-05	4.85E-03	*ITGAM; IL23R; IRF1; CD58; TYK2;* *JAK2; FGF2; PTPN2; IL22RA2*
Regulation of tyrosine phosphorylation of STAT protein (GO:0042509)	5.54E-05	7.40E-03	*IL23R; JAK2; PTPN2; IL22RA2*
**Term (GO_molecular_function)**	***P*****-value**	**Adjusted** ***P*****-value**	**Genes**
Growth hormone receptor binding (GO:0005131)	3.06E-04	1.98E-02	*TYK2; JAK2*
Kinase activity (GO:0016301)	3.55E-04	1.98E-02	*PRKAA1; DGKQ; TNIK; JAK2; MAP3K7; MAP4K4*
Protein kinase activity (GO:0004672)	3.11E-04	1.98E-02	*PRKAA1; MST1R; TNIK; TYK2;* *JAK2; FGF2; MAP3K7; MAP4K4*
MAP kinase kinase kinase kinase activity (GO:0008349)	7.14E-04	2.98E-02	*TNIK; MAP4K4*
Phosphotransferase activity, alcohol group as acceptor (GO:0016773)	1.64E-03	4.58E-02	*PRKAA1; DGKQ; TNIK; JAK2; MAP3K7*

### Protein-Protein Interaction Network Analysis in String

**Figure 2:**
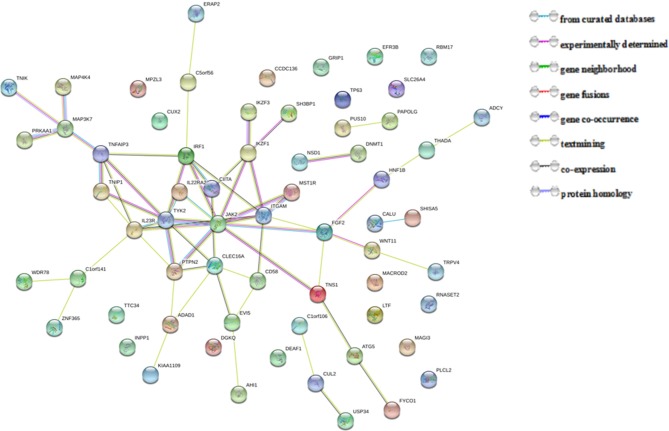
Protein-protein interactions between 67 pleiotropic genes associated with seven autoimmune/autoinflammatory diseases.

The 67 putative pleiotropic genes identified were retrieved from the STRING database. Of these, 63 genes were clearly enriched in two confirmed clusters: *JAK2* and *MAP3K7* clusters (Figure 2). Three of the novel putative pleiotropic genes detected, *FGF2, IL22RA2*, and *ITGAM*, are involved in the *JAK2* cluster, which participates in various processes such as cell growth, development, differentiation or histone modifications, and mediates essential signaling events in both innate and adaptive immunity. Three other novel genes, *MAP4K4, PRKAA1*, and *TNIK*, were involved in the *MAP3K7* cluster, which acts as an essential component of the MAP kinase signal transduction pathway and plays a role in the response to environmental stress and cytokines such as TNF-alpha.

## Discussion

In the present study, a novel analytical approach—metaCCA—was used to explore the common genetic variants associated with autoimmune/autoinflammatory diseases by combining seven available independent GWAS or meta-analysis summary statistics. A total of 67 putative pleiotropic genes were successfully identified after verification using gene-based analysis. In particular, 27 confirmed genes were identified as pleiotropic in previous different types of studies and were validated in the present study, 16 novel pleiotropic genes were previously reported to be associated with one autoimmune/autoinflammatory disease, and 24 candidate novel pleiotropic genes that had never been reported to be associated with any autoimmune/autoinflammatory disease. The improved detection of pleiotropic genes and the associated biological pathways may provide novel insights into the shared genetic factors involved in development of autoimmune/autoinflammatory diseases.

Among the 27 confirmed pleiotropic genes, 6 genes (*ADAD1, CIITA, CLEC16A, IL2*3R, *MAGI3*, and *PTPN2*), which play an important role on the pathomechanism of autoimmune/autoinflammatory diseases, were shown to be associated with more than one autoimmune/autoinflammatory disease not only in the literature review, but also in the VEGAS2 analysis using original GWAS summary statistics. For example, common genetic variants of *CLEC16A*, also known as C-type lectin-like domain family 16A, had been reported to be associated with CEL, IBD, MS, PBC, and T1D (10). As the non-HLA genome-wide significant risk gene, *CLEC16A* is essential for autophagosomal growth and autophagy processes, which are of major importance for proper immune regulation, including regulation of inflammasome activation (39, 40). Moreover, recent data from murine studies and our PPIs analysis indicated that *CLEC16A* plays a key role in beta cells functions by regulating mitophagy/autophagy and mitochondrial health (41). *PTPN2* is another important and confirmed pleiotropic gene associated with several autoimmune/autoinflammatory diseases we studied (10). The GO term enrichment analysis results suggested that *PTPN2* encodes T-cell protein tyrosine phosphatase, acting as a negative regulator of the JAK/STAT signaling pathways downstream of cytokines and playing a prominent role in T-cell activation, signaling and/or effector function, which may represent potential targets for the pharmacotherapy of autoimmune diseases. In addition, Mei et al. (42) also showed that, in the Northeastern Chinese population, *PTPN2* polymorphisms are associated with psoriasis, which is another chronic immune-mediated disease with a complex etiology.

Sixteen novel putative pleiotropic genes detected in this study had previously been confirmed to be associated with one form of autoimmune/autoinflammatory disease. Interestingly, *EFR3B* and *RBM17* had been reported to be associated only with T1Din published studies, but were confirmed to be associated with other diseases in the VEGAS2 analysis (13, 43–45). *EFR3B* is an associated gene located in 2p23, which probably acts as the membrane-anchoring component and is involved in responsiveness to G-protein-coupled receptors. Although Bradfield et al. (13) have confirmed that *EFR3B* is an associated loci and protein-protein interaction network analysis also provided some protein information, it is still unclear whether this role is direct or indirect. *RBM17*, which is involved in the regulation of alternative splicing and the utilization of cryptic splice sites, is essential for survival and cell maintenance. Fortunately, genetic and serologic data suggest that the inherited altered genetic constitution located between *IL2RA* and *RBM17* may predispose to a less destructive course of RA (46, 47). Although the 14 remaining genes were identified associated with one form of autoimmune/autoinflammatory disease in the literature review and VEGAS2 analysis, further experimental studies are required to confirm their role as pleiotropic genes associated with autoimmune/autoinflammatory diseases. *CUL2*, which is associated with response to the hypoxic environment and activation of tumor immunity, has been identified in association with CRO in nine independent case-control series (48). Zhang et al. (49) suggested that human immunodeficiency virus type 1 and simian immunodeficiency virus viral infectivity factor form a CRL5 E3 ubiquitin ligase complex that suppresses virus restriction by host APOBEC3 proteins, and that *CUL2* eventually suppresses this pathway and increases the risk of autoimmune/autoinflammatory diseases (50).

Significantly, nine genes (*C1orf141, CALU, CCDC136, FGF2, LOC101927051, MAP4K4, MPZL3, PAPOLG*, and *SH3BP1*) were found to be associated with more than one disease in the VEGAS2 analysis, although these genes had never been reported to be associated with any autoimmune/autoinflammatory disease in previous GWAS. *MAP4K4* has been enriched in several GO terms including MAP kinase c kinase activity (GO:0008349), which is an important contributor to the risk of developing type 2 diabetes mellitus in a Chinese Han population (51). In addition, Aouadi et al. (52) demonstrated that orally delivered small interfering RNA targeting macrophage *MAP4K4* suppresses systemic inflammation, thus implicating this technology as a new strategy to attenuate inflammatory responses in human disease. *C1orf141* is another significant candidate novel pleiotropic gene found to be associated with IBD and PBC in our study. It has been recently shown that *C1orf141* is a susceptibility variant in psoriasis, a chronic inflammatory hyperproliferative cutaneous disease (53). Further studies are required to confirm our findings and provide a detailed description of each candidate novel gene and the associated pathomechanism.

Systematic and comprehensive searches for pleiotropic genes and their effects are essential for an understanding of the mechanisms underlying the development of autoimmune/autoinflammatory diseases (4). Compared to the univariate GWAS analysis based on a cross-sectional population, our study was cost-effective and reliable, not only due to the increased sample size achieved by integrating the summary statistics of seven large GWAS. This approach also increased the statistical power of the study and provided a wealth of information by the simultaneous analysis of multiple related autoimmune/autoinflammatory diseases. However, because of a lack of detailed original individual measures, we were unable to determine whether the effects of pleiotropic genes on risk of these diseases are direct or indirect. Alternative approaches and experimental studies are required to validate these novel genes identified in this study.

In summary, we have provided convincing evidence of the existence of 27 confirmed pleiotropic genes and highlighted 40 novel pleiotropic genes associated with autoimmune/autoinflammatory diseases by performing a systematic multivariate analysis of the open GWAS data using metaCCA. Furthermore, we have illustrated potential biological functions of these pleiotropic genes and our results contribute to a better understanding of common genetic mechanisms, and eventually the development of improved diagnosis, prognosis and targeted therapies.

## Data Availability Statement

All datasets generated for this study are included in the article.

## Author Contributions

XJ, YY, and XS conceived and designed the study, analyzed the data, and wrote the manuscript. YY and FX performed the methodology and data curation. XJ, NS, YF, JT, and YL analyzed the data. XS, WW, CS, and HD contributed to the critical revision of the manuscript.

### Conflict of Interest

The authors declare that the research was conducted in the absence of any commercial or financial relationships that could be construed as a potential conflict of interest.

## References

[1] Brooks WH. Involvement of X chromosome short arm in autoimmune diseases: comment on the article by Sharma et al. Arthr Rheumatol. (2018) 70:625–6. 10.1002/art.40411 29316369

[2] Cooper GS Bynum MLK Somers EC. Recent insights in the epidemiology of autoimmune diseases: improved prevalence estimates and understanding of clustering of diseases. J Autoimmun. (2009) 33:197–207. 10.1016/j.jaut.2009.09.008 19819109 PMC2783422

[3] Ludwig RJ Karen V Frank L Ziya K Katja B MS M. Mechanisms of autoantibody-induced pathology. Front Immunol. (2017) 8:603. 10.3389/fimmu.2017.00603 28620373 PMC5449453

[4] Cotsapas C Hafler DA. Immune-mediated disease genetics: the shared basis of pathogenesis. Trends Immunol. (2013) 34:22–6. 10.1016/j.it.2012.09.001 23031829

[5] Cotsapas C Voight BF Rossin E Lage K Neale BM Wallace C. Pervasive sharing of genetic effects in autoimmune disease. PLoS Genet. (2011) 7:e1002254. 10.1371/journal.pgen.1002254 21852963 PMC3154137

[6] Prüßmann J Prüßmann W Recke A Rentzsch K Ludwig RJ. Co-occurrence of autoantibodies in healthy blood donors. Exp Dermatol. (2014) 23:519–21. 10.1111/exd.12445 24816528

[7] Stearns FW One hundred years of pleiotropy: a retrospective (vol 186, pg 767, 2010). Genetics. (2011) 187:355 10.1534/genetics.110.122549 PMC2975297 21062962

[8] Armstrong DL Zidovetzki R Alarcon-Riquelme ME Tsaos BP Criswell LA Kimberly RP. GWAS identifies novel SLE susceptibility genes and explains the association of the HLA region. Genes Immun. (2014) 15:347–54. 10.1038/gene.2014.23 24871463 PMC4156543

[9] Sirota M Schaub MA Batzoglou S Robinson WH Butte AJ. Autoimmune disease classification by inverse association with SNP alleles. PLoS Genet. (2009) 5:e1000792. 10.1371/journal.pgen.1000792 20041220 PMC2791168

[10] Li YR Li J Zhao SD Bradfield JP Mentch FD Maggadottir SM. Meta-analysis of shared genetic architecture across ten pediatric autoimmune diseases. Nat Med. (2015) 21:1018–27. 10.1038/nm.3933 26301688 PMC4863040

[11] Winkler TW Day FR Croteau-Chonka DC Wood AR Locke AE Maegi R. Quality control and conduct of genome-wide association meta-analyses. Nat Protoc. (2014) 9:1192–212. 10.1038/nprot.2014.071 24762786 PMC4083217

[12] Guerini FR Bolognesi E Manca S Sotgiu S Zanzottera M Agliardi C. Family-based transmission analysis of HLA genetic markers in Sardinian children with autistic spectrum disorders. Hum Immunol. (2009) 70:184–90. 10.1016/j.humimm.2008.12.009 19167444

[13] Bradfield JP Qu H-Q Wang K Zhang H Sleiman PM Kim CE. A genome-wide meta-analysis of six type 1 diabetes cohorts identifies multiple associated Loci. PLoS Genet. (2011) 7:e1002293. 10.1371/journal.pgen.1002293 21980299 PMC3183083

[14] Tang CS Ferreira MAR. A gene-based test of association using canonical correlation analysis. Bioinformatics. (2012) 28:845–50. 10.1093/bioinformatics/bts051 22296789

[15] Kettunen J Tukiainen T Sarin A-P Ortega-Alonso A Tikkanen E Lyytikainen L-P. Genome-wide association study identifies multiple loci influencing human serum metabolite levels. Nat Genet. (2012) 44:269–76. 10.1038/ng.1073 22286219 PMC3605033

[16] Inouye M Ripatti S Kettunen J Lyytikainen L-P Oksala N Laurila P-P. Novel Loci for metabolic networks and multi-tissue expression studies reveal genes for atherosclerosis. PLoS Genet. (2012) 8:e1002907. 10.1371/journal.pgen.1002907 22916037 PMC3420921

[17] Evangelou E Ioannidis JPA. Meta-analysis methods for genome-wide association studies and beyond. Nat Rev Genet. (2013) 14:379–89. 10.1038/nrg3472 23657481

[18] Chung D Yang C Li C Gelernter J Zhao H. GPA: A statistical approach to prioritizing GWAS results by integrating pleiotropy and annotation. PLoS Genet. (2014) 10:e1004787. 10.1371/journal.pgen.1004787 25393678 PMC4230845

[19] Cichonska A Rousu J Marttinen P Kangas AJ Soininen P Lehtimaki T. metaCCA: summary statistics-based multivariate meta-analysis of genome-wide association studies using canonical correlation analysis. Bioinformatics. (2016) 32:1981–9. 10.1093/bioinformatics/btw052 27153689 PMC4920109

[20] Jia X Yang Y Chen Y Cheng Z Du Y Xia Z. Multivariate analysis of genome-wide data to identify potential pleiotropic genes for five major psychiatric disorders using MetaCCA. J Affect Disord. (2019) 242:234–43. 10.1016/j.jad.2018.07.046 30212762 PMC6343670

[21] Jia X Yang Y Chen Y Xia Z Zhang W Feng Y. Multivariate analysis of genome-wide data to identify potential pleiotropic genes for type 2 diabetes, obesity and coronary artery disease using MetaCCA. Int J Cardiol. (2019) 283:144–50. 10.1016/j.ijcard.2018.10.102. 30459114

[22] Chen Y-C Xu C Zhang J-G Zeng C-P Wang X-F Zhou R. Multivariate analysis of genomics data to identify potential pleiotropic genes for type 2 diabetes, obesity and dyslipidemia using Meta-CCA and gene-based approach. PLoS ONE. (2018) 13:e0201173. 10.1371/journal.pone.0201173 30110382 PMC6093635

[23] Dubois PCA Trynka G Franke L Hunt KA Romanos J Curtotti A. Multiple common variants for celiac disease influencing immune gene expression. Nat Genet. (2010) 42:295–302. 10.1038/ng.543 20190752 PMC2847618

[24] de lange KM Moutsianas L Lee JC Lamb CA Luo Y Kennedy NA Genome-wide association study implicates immune activation of multiple integrin genes in inflammatory bowel disease. Nat Genet. (2017) 49:256–61. 10.1038/ng.3760 28067908 PMC5289481

[25] Sawcer S Hellenthal G Pirinen M Spencer CCA Patsopoulos NA Moutsianas L. Genetic risk and a primary role for cell-mediated immune mechanisms in multiple sclerosis. Nature. (2011) 476:214–9. 10.1038/nature10251 21833088 PMC3182531

[26] Cordell HJ Han Y Mells GF Li Y Hirschfield GM Greene CS. International genome-wide meta-analysis identifies new primary biliary cirrhosis risk loci and targetable pathogenic pathways. Nat Commun. (2015) 6:8019. 10.1038/ncomms9019 26394269 PMC4580981

[27] Stahl EA Raychaudhuri S Remmers EF Xie G Eyre S Thomson BP. Genome-wide association study meta-analysis identifies seven new rheumatoid arthritis risk loci. Nat Genet. (2010) 42:508–14. 10.1038/ng.582 20453842 PMC4243840

[28] Bentham J Morris DL Graham DSC Pinder CL Tombleson P Behrens TW. Genetic association analyses implicate aberrant regulation of innate and adaptive immunity genes in the pathogenesis of systemic lupus erythematosus. Nat Genet. (2015) 47:1457–64. 10.1038/ng.3434 26502338 PMC4668589

[29] Censin JC Nowak C Cooper N Bergsten P Todd JA Fall T. Childhood adiposity and risk of type 1 diabetes: a Mendelian randomization study. PLoS Med. (2017) 14: e1002362. 10.1371/journal.pmed.1002362 PMC5538636 28763444

[30] Zhang Q Wu K-H He J-Y Zeng Y Greenbaum J Xia X. Novel common variants associated with obesity and type 2 diabetes detected using a cFDR method. Sci Rep. (2017) 7:16397. 10.1038/s41598-017-16722-6 29180724 PMC5703959

[31] Seoane JA Campbell C Day INM Casas JP Gaunt TR. Canonical correlation analysis for gene-based pleiotropy discovery. PLoS Comput Biol. (2014) 10:e1003876. 10.1371/journal.pcbi.1003876 25329069 PMC4199483

[32] Mishra A Macgregor S. VEGAS2: software for more flexible gene-based testing. Twin Res Hum Genet. (2015) 18:86–91. 10.1017/thg.2014.79 25518859

[33] Wojcik GL Kao WHL Duggal P. Relative performance of gene- and pathway-level methods as secondary analyses for genome-wide association studies. BMC Genet. (2015) 16:34. 10.1186/s12863-015-0191-2 25887572 PMC4391470

[34] Lv W-Q Zhang X Zhang Q He J-Y Liu H-M Xia X. Novel common variants associated with body mass index and coronary artery disease detected using a pleiotropic cFDR method. J Mol Cell Cardiol. (2017) 112:1–7. 10.1016/j.yjmcc.2017.08.011 28843344 PMC5812278

[35] Chen EY Tan CM Kou Y Duan Q Wang Z Meirelles GV. Enrichr: interactive and collaborative HTML5 gene list enrichment analysis tool. BMC Bioinformatics. (2013) 14:128. 10.1186/1471-2105-14-128 23586463 PMC3637064

[36] Zhang G Zhang W. Protein-protein interaction network analysis of insecticide resistance molecular mechanism in *Drosophila melanogaster*. Arch Insect Biochem Physiol. (2019) 100:e21523. 10.1002/arch.21523 30478906

[37] Fierabracci A Milillo A Locatelli F Fruci D. The putative role of endoplasmic reticulum aminopeptidases in autoimmunity: Insights from genomic-wide association studies. Autoimmun Rev. (2012) 12:281–8. 10.1016/j.autrev.2012.04.007 22575366

[38] Szklarczyk D Franceschini A Wyder S Forslund K Heller D Huerta-Cepas J. STRING v10: protein-protein interaction networks, integrated over the tree of life. Nucleic Acids Res. (2015) 43:D447–52. 10.1093/nar/gku1003 25352553 PMC4383874

[39] Liu JZ Almarri MA Gaffney DJ Mells GF Jostins L Cordell HJ Dense fine-mapping study identifies new susceptibility loci for primary biliary cirrhosis. Nat Genet. (2012) 44:1137–41. 10.1038/ng.2395 22961000 PMC3459817

[40] Berge T Leikfoss IS Harbo HF. From identification to characterization of the multiple sclerosis susceptibility gene CLEC16A. Int J Mol Sci. (2013) 14:4476–97. 10.3390/ijms14034476 23439554 PMC3634488

[41] Li J Jorgensen SF Maggadottir SM Bakay M Warnatz K Glessner J. Association of CLEC16A with human common variable immunodeficiency disorder and role in murine B cells. Nat Commun. (2015) 6:6804. 10.1038/ncomms7804 25891430 PMC4444044

[42] Mei Q Liu C Zhang X Li Q Jia X Wu J. Associations between PTPN2 gene polymorphisms and psoriasis in Northeastern China. Gene. (2019) 681:73–9. 10.1016/j.gene.2018.09.047 30266502

[43] Oldstone MBA Edelmann KH Mcgavern DB Cruite JT Welch MJ. Molecular anatomy and number of antigen specific CD8 T cells required to cause type 1 diabetes. PLoS Pathog. (2012) 8:1352–62. 10.1371/journal.ppat.1003044 23209415 PMC3510245

[44] Honke N Shaabani N Zhang DE Iliakis G Xu HC Häussinger D. Usp18 driven enforced viral replication in dendritic cells contributes to break of immunological tolerance in autoimmune diabetes. PLoS Pathog. (2013) 9:e1003650. 10.1371/journal.ppat.1003650 24204252 PMC3812017

[45] Sharma A Liu X Hadley D Hagopian W Chen WM Onengut-Gumuscu S. Identification of non-HLA genes associated with development of islet autoimmunity and type 1 diabetes in the prospective TEDDY cohort. J Autoimmun. (2018) 89:90–100. 10.1016/j.jaut.2017.12.008 29310926 PMC5902429

[46] Knevel R de Rooy DPC Zhernakova A Grondal G Krabben A Steinsson K. Association of variants in IL2RA with progression of joint destruction in rheumatoid arthritis. Arthr Rheum. (2013) 65:1684–93. 10.1002/art.37938 23529819

[47] Steer S Abkevich V Gutin A Cordell HJ Gendall KL Merriman ME. Genomic DNA pooling for whole-genome association scans in complex disease: empirical demonstration of efficacy in rheumatoid arthritis. Genes Immun. (2007) 8:57–68. 10.1038/sj.gene.6364359 17159887

[48] Rivas MA Beaudoin M Gardet A Stevens C Sharma Y Zhang CK. Deep resequencing of GWAS loci identifies independent rare variants associated with inflammatory bowel disease. Nat Genet. (2011) 43:1066–73. 10.1038/ng.952 21983784 PMC3378381

[49] Zhang W Wang H Li Z Liu X Liu G Harris RS. Cellular requirements for Bovine immunodeficiency virus Vif-mediated inactivation of Bovine APOBEC3 proteins. J Virol. (2014) 88:12528–40. 10.1128/JVI.02072-14 25142583 PMC4248920

[50] Brooks JM Long HM Tierney RJ Shannon-Lowe C Leese AM Fitzpatrick M. Early T cell recognition of B cells following Epstein-Barr virus infection: identifying potential targets for prophylactic vaccination. PLoS Pathog. (2016) 12:e1005549. 10.1371/journal.ppat.1005549 27096949 PMC4838210

[51] Li T-T Qiao H Tong H-X Zhuang T-W Wang T-T. Association of common genetic variants in mitogen-activated Protein Kinase Kinase Kinase Kinase 4 with Type 2 Diabetes Mellitus in a Chinese Han Population. Chin Med J. (2016) 129:1179–84. 10.4103/0366-6999.181969 27174326 PMC4878163

[52] Aouadi M Tesz GJ Nicoloro SM Wang M Chouinard M Soto E. Orally delivered siRNA targeting macrophage Map4k4 suppresses systemic inflammation. Nature. (2009) 458:1180–4. 10.1038/nature07774 19407801 PMC2879154

[53] Zuo X Sun L Yin X Gao J Sheng Y Xu J. Whole-exome SNP array identifies 15 new susceptibility loci for psoriasis. Nat Commun. (2015) 6:6793. 10.1038/ncomms7793 25854761 PMC4403312

[54] Jia X Shi N Xia Z Feng Y Li Y Tan J Identification of 67 pleiotropic genes for seven autoimmune diseases using multivariate statistical analysis. bioRxiv [Preprint]. 10.1101/563973 PMC7008725 32117227

